# Effect of Gender, Age, and Ocular and Growth-Related Factors on Corneal Epithelial and Stromal Thickness in Children

**DOI:** 10.3390/jcm9123849

**Published:** 2020-11-27

**Authors:** Wook Kyum Kim, Ik Hee Ryu, Jeongseo Yoo, Sun Woong Kim

**Affiliations:** 1 B & VIIT Eye Center, Seoul 06615, Korea; kiki0306@daum.net (W.K.K.); ikheeryu@gwbnviit.com (I.H.R.); 2 Department of Ophthalmology, Yonsei University Wonju College of Medicine, Wonju 26426, Gangwon-do, Korea; yjs940602@naver.com

**Keywords:** corneal epithelial thickness, corneal stromal thickness, corneal thickness measurement, myopia, keratoconus, anterior segment optical coherence tomography

## Abstract

Data on corneal epithelial and stromal thickness in school-aged children in relation to gender, age, and ocular and growth parameters are limited. In this retrospective study, we analyzed corneal epithelial and stromal thickness measured with the RTVue system (Optovue, Inc., Fremont, CA, USA) in 122 male and 201 female Korean children (mean age 9.59 ± 2.18 years) with myopia. We used simple and multiple regression analysis to establish the relationships between gender, age, refractive status, axial length, anterior chamber depth (ACD), corneal refractive power, white-to-white corneal diameter (WTW), height, and body weight. Age, body weight, height, and central corneal thickness were positively associated with corneal epithelial thickness, whereas WTW was negatively associated. The multiple regression analysis showed corneal epithelial thickness was affected by sex, body weight, WTW, and central corneal thickness (CCT), while stromal thickness was influenced by age, sex, and WTW. Both corneal epithelial and stromal thickness were significantly greater in male than in female children and were affected by growth. Neither corneal epithelial nor stromal thickness were associated with the severity of myopia, corneal refractive power, or axial length.

## 1. Introduction

The measurement of corneal thickness plays a supportive role in various clinical decisions, such as determining patients’ eligibility for refractive surgery, the diagnosis of keratoconus or corneal edema, and providing essential references in the assessment of intraocular pressure in patients with glaucoma [1,2,3,4]. Recent developments in topographic epithelial thickness mapping, using optical coherence tomography, have drawn increasing attention and suggest many important clinical applications. These developments enable the rapid measurement of corneal epithelial thickness in daily routine with good repeatability but without contact. Previous studies reported good repeatability of corneal thickness and epithelial thickness measurements in normal eyes and eyes after myopic refractive surgery using the RTVue system (Optovue, Inc., Fremont, CA, USA) [5,6,7].

Regarding the plasticity of the epithelium, its thickness profile could have various clinical implications. For example, epithelial thickness adapts to reduce stromal irregularities in diseases like keratoconus. Hence, the ability to analyze corneal epithelial and stromal thicknesses and shapes separately may be useful for early diagnosis of disease [8,9,10]. Furthermore, knowledge of the epithelial thickness profile and how it changes after surgery may positively contribute to the refractive outcome and be useful in planning refractive surgery [11,12]. Central epithelial hyperplasia has been associated with myopic regression after laser refractive surgery, and epithelial remodeling has been suggested as a treatment strategy [13,14,15,16]. Moreover, corneal epithelial thickness profiles could be useful in school-aged children because central epithelial thinning has been associated with the required magnitude of myopia correction in orthokeratology [17,18,19]. As orthokeratology corrects myopia through epithelial remodeling, epithelial thickness profiles may assist in the modification of lens design [19,20]. Consequently, corneal epithelial thickness maps taken from normal children might provide references to identify abnormal values, help to assess the eligibility, and predict the response to orthokeratology. 

When assessing corneal epithelial or stromal thickness, the effects of race, gender, age, and refractive status have been widely studied. The influence of gender has been well documented, but results on the influence of age on corneal epithelial thickness have not been consistent [21,22,23,24]. Moreover, the influence of a refractive error on corneal thickness, especially epithelial thickness, has not been clearly established to date [25,26,27,28,29]. Although epithelial thickness profiles have been published broadly, there are limited data on school-aged children [30].

In this study, we aimed to investigate the influence of gender, age, refraction, corneal refractive power, and various ocular and growth-related measurements on corneal epithelial and stromal thicknesses in Korean children. This study will provide a normative database for school-aged children and help us understand the change in corneal epithelial and stromal thickness with age for the first time.

## 2. Materials and Methods

This retrospective cohort study included 375 Korean children who came to B & VIIT Eye Center mostly for annual check-ups, glasses prescriptions, contact lenses and whom were evaluated for corneal thickness measurement between February 2017 and April 2020. Based on the data of their complete ophthalmologic examinations, including slit-lamp examination, intraocular pressure, corneal thickness, manifest refraction, topography, and fundus examination, we excluded 52 subjects with ocular pathologies such as corneal opacity, corneal dystrophies, keratoconus, a history of contact lens wearing in the previous 4 weeks, glaucoma, and prior ocular surgery. Consequently, we analyzed 323 children in the study.

In addition, we recorded the data for mean corneal power, axial length, anterior chamber depth (ACD), and white-to-white corneal diameter (WTW) obtained using an AL-scan (Nidek Co., Ltd., Tokyo, Japan). Furthermore, body height and weight measurements using an InBody J50 (InBody Co., Ltd., Seoul, Korea) were recorded in 165 children. 

Corneal epithelial and total thickness data were obtained using the RTVue Fourier-domain optical coherence tomography (FD-OCT) system (Optovue, Inc., Fremont, CA, USA) with a corneal adaptor module at 830 nm wavelength. The system generates corneal epithelial thickness maps using an automatic algorithm divided into a total of 17 sectors: a central 2 mm diameter zone, eight paracentral zones within the annulus between 2 and 5 mm diameter rings, and eight mid-peripheral zones within the annulus between 5 and 6 mm diameter rings. The stromal thickness was calculated by subtracting epithelial thickness from the corneal thickness for each area. 

The study protocol was approved by the institutional review board of our institute (CR320133), and all procedures followed the rules of the Declaration of Helsinki.

The corneal thickness and ocular measurements were highly correlated between the right and left eyes, and all measurement data of the right eye of each subject were analyzed to reduce an increased risk of a type 1 error. The statistical analyses were performed using IBM SPSS Statistics for Windows, version 25.0 (IBM Corp., Armonk, NY, USA). Student’s *t*-test was used to compare continuous variables between male and female children. Simple linear regression was used to determine a potential association between age, sex, refractive error (spherical equivalent refraction), axial length, ACD, mean corneal refractive power (mean K), WTW, height, body weight and corneal epithelial and stromal thickness. Multiple linear regression analysis was conducted to investigate the association of factors identified during simple regression analysis with corneal epithelial and stromal thickness. Pearson correlation analysis was used to investigate the association between ocular and growth-related factors. Statistical significance was defined as a *p*-value < 0.05.

## 3. Results

### 3.1. Corneal Epithelial and Stromal Thickness Profiles

This study analyzed 323 right eyes of 323 Korean children (122 male and 201 female) with a mean age of 9.59 ± 2.18 years (range 6–17 years). There was a statistically significant difference in the mean age between male and female children, but comparison between male and female would be acceptable as the difference is small in amount (9.16 and 9.84 years, respectively). In our cohort, the mean K (corneal refractive power) was greater, and the corneal epithelial and stromal thickness was less in female than those in male children (Table 1).

**Figure 1:**
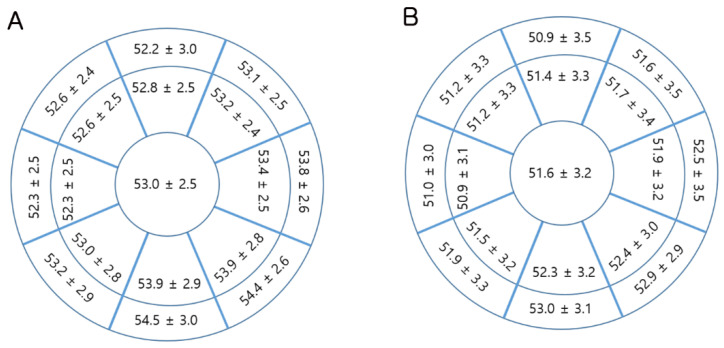
Topographic corneal epithelial thickness map for (**A**) male children (*n* = 122) and (**B**) female children (*n* = 201).

In the sub-analysis of 165 children with weight and height measurements, a similar pattern was observed for age, mean K, and corneal thickness, although the difference in stromal thickness was not statistically significant. The axial length was greater in male than in female children, whereas ACD and WTW were similar. In this subgroup, body growth measurements were similar between male and female children (Table 2). The refractive status between male and female children was not different. Although the difference in the mean corneal epithelial thickness between male and female children was small, it was a consistent finding in all 17 sections within the 6.0 mm diameter (Figure 1).

Simple regression analysis indicated that corneal epithelial thickness was positively associated with sex, age, central corneal thickness (CCT), height, and weight, while it showed a negative linear relationship with the WTW. Multiple regression indicated that sex, body weight, WTW, and CCT were significant factors affecting corneal epithelial thickness in the central 2 mm zone (Table 3). In the paracentral zone, sex, body weight, WTW, and CCT were significant factors, and in the mid-peripheral zone, sex and body weight were significant factors affecting epithelial thickness (Table 4). Older age, male gender, and a smaller WTW were associated with greater corneal stromal thickness in the central, paracentral, and mid-peripheral zones.

### 3.2. Association between Ocular Measurements

The severity of myopia was correlated with greater axial length, ACD, height, body weight, and age. Mean corneal power did not show an association with age and was inversely proportional to the axial length and WTW (Table 5). WTW was inversely correlated with mean corneal power, CCT, and epithelial/stromal thickness and proportional to the axial length and ACD.

## 4. Discussion

There are many demographic, ethnic, and ophthalmologic factors that potentially affect corneal thickness [31,32,33,34]. Some studies reported no correlation of CCT with age [22,27], but others indicated its decrease with age [31,32,33,34]. Similarly, a gender difference was not always identified, but many researchers reported a thicker cornea in males than in females in adults [28,33]. This difference may be attributed to the endocrine differences between males and females [35,36]. The recent availability of corneal epithelial imaging using FD-OCT allows for in vivo epithelial mapping. However, there are few reports investigating possible factors affecting corneal epithelial thickness. An age-related decrease in corneal epithelial thickness has been reported, especially in the peripheral and limbal area in adults, but there were no significant differences in the center [23,24]. In our previous work, we reported that gender is correlated with epithelial thickness, while the severity of myopia is negatively associated with stromal thickness [29].

Corneal epithelial thickness has drawn increasing attention in children because it manifests structural and functional changes under various conditions [19,30]. This study is the first to investigate corneal epithelial and stromal thickness in Korean children. Overall, topographic corneal epithelial thickness profiles were similar to adult profiles: superior and temporal epithelial thickness is lower than inferior and nasal epithelial thickness, respectively, as described in previous reports [5,23]. Interestingly, stromal thickness showed opposite regional differences: the superior stroma is thicker than the inferior stroma. Males showed a thicker cornea, especially of the epithelium, than females. A recent publication by Ma et al. [30] reported that a thicker central corneal epithelium was associated with male gender and older age but not with the corneal curvature radius, axial length, or refraction in Chinese school-aged children. The previous research reported that age, gender, and corneal curvature radius were correlated with paracentral and mid-peripheral epithelial thickness.

Our group has monitored the change in corneal epithelial thickness during the follow-up of orthokeratology for myopic correction in school-aged children previously [19]. In the present study, corneal epithelial thickness appeared to increase with age, a similar finding to another study [30]. The linear association between age and corneal epithelial thickness remained after controlling for gender. The age dependence and gender difference of corneal epithelial thickness inspired us to explore the effect of growth. Interestingly, the influence of age on epithelial thickness was insignificant when body weight and WTW corneal diameter were included in the multiple regression analysis. A thicker corneal epithelium was associated with male gender, greater body weight and height, a smaller corneal diameter, and greater central corneal thickness. This result shows that the linear association between age and corneal epithelial thickness is due to the linear association with overall growth. Ocular factors such as refractive error, mean K, ACD, and axial length were not associated with corneal epithelial or stromal thickness. A thicker corneal stroma appeared to be associated with age, male gender, and a small corneal diameter. The gender difference in corneal epithelial and stromal thickness was consistent and remained significant after controlling for growth-related variables. As such, even if of small magnitude, the gender difference should be considered. It is noteworthy that the gender difference was more prominent for epithelial thickness than stromal thickness. Our findings support a previous report that gonadal hormones may affect ocular tissue growth [35]. Different from previous findings, there was no association between epithelial thickness and corneal power. We cannot explain this difference, and this needs to be further investigated. 

To understand age-related changes in epithelial thickness, we compared our current results with those from our previous publication [23]. Taking the growth-associated increase in epithelial thickness in children into consideration, the difference in the mean central epithelial thickness between children and adults (52.1 vs. 53.8 [23]) may be not surprising. The corneal epithelium of children was thinner than that of young adults (18–29 years) and thicker than that of old adults (60–80 years) in the paracentral and mid-peripheral area. Considering our findings and the results of previous studies, the corneal epithelium seems to become thicker during childhood, remain stable during early adulthood but become thinner at old age, especially in the periphery.

The strengths of this study include the exploration of the association between corneal sublayer thickness and widely used ocular measurements together with growth-related variables. It is noteworthy that males showed greater axial length and lower mean corneal power than females, potentially explaining the similar degree of myopia among male and female children in our study. Although our study participants may not be representative of Korean children in general, our data provide insight into the relationships between ocular and growth-related parameters. A previous study reported that the refractive error and CCT were inversely proportional to mean corneal power (r = −0.18 and −0.11, respectively) in myopic patients [37]. Another study found that corneal power negatively correlated with the axial length, ACD, and WTW [38]. Our data showed that mean corneal power was inversely proportional to the axial length and WTW, but axial length did not show a linear correlation with corneal epithelial thickness or CCT. The absence of a correlation between the refractive error and mean corneal power or CCT in the current study may have resulted from differences in participants’ age and sample size. 

Our data revealed that WTW as an ocular variable is correlated with many other measurements. It is inversely correlated with mean corneal power, CCT, and epithelial/stromal thickness and proportional to axial length and ACD. The clinical implications of different WTW measurements should be studied further. Overall, our data suggest that corneal power tends to decrease as axial length increases during childhood growth. 

There are several limitations to our study. Firstly, this was a retrospective study and only included a limited range of refractive errors from +0.75 to −6 D in spherical errors and +0.5 to −1.75 D in cylindrical errors. Since this study analyzed hospital-based data, the refractive errors of subjects were mostly mild to moderate myopia. Considering the large population of myopic subjects of East Asian ethnicity, this is not unexpected, but we admit that our results may not be representative of Korean children in general. The absence of a correlation between stromal thickness and severity of myopia may result from this limited range of refractive errors. Secondly, the corneal epithelial thickness measured by FD-OCT includes the tear film thickness, which might overestimate its true value. This technical issue has been described previously [23,24], but the high repeatability, non-invasiveness, and easy acquisition of measurements make this device very helpful in the clinic and may be advantageous, especially in children.

## 5. Conclusions

Our study presents corneal epithelial thickness profiles for Korean children aged 6 to 17 years. Corneal epithelial thickness showed a similar topographic profile to that of adults, and male children had a thicker epithelium than female children. Corneal epithelial thickness was affected by gender, CCT, WTW, and body weight, while stromal thickness was affected by age, gender, and WTW. Neither corneal epithelial nor stromal thickness were associated with the severity of myopia, corneal refractive power, or axial length.

## Figures and Tables

**Table 1:** Corneal epithelial and stromal thickness in Korean school children (*n* = 323).

	Male (*n* = 122)	Female (*n* = 201)	*p*-Value
Age (years)	9.16 ± 2.04	9.84 ± 2.23	0.006 *
SEQ (D)	−2.29 ± 1.40	−2.40 ± 1.34	0.468
CCT (µm)	540.43 ± 32.77	531.37 ± 32.10	0.015 *
Mean K (D)	43.01 ± 1.33	43.64 ± 1.22	<0.001 *
Epithelium (µm)			
Central	52.98 ± 2.50	51.57 ± 3.22	<0.001 *
Paracentral	53.14 ± 2.36	51.67 ± 3.02	<0.001 *
Midperiphery	53.79 ± 6.02	51.88 ± 2.81	<0.001 *
Stroma (µm)			
Central	487.45 ± 32.24	479.80 ± 31.57	0.037 *
Paracentral	507.30 ± 32.75	498.95 ± 32.34	0.026 *
Midperiphery	528.80 ± 34.34	521.29 ± 32.79	0.051

* Statistically significant difference at *p* < 0.05, Student’s *t*-test. Values are presented as the mean ± standard deviation (SD). SEQ, spherical equivalent refraction; CCT, central corneal thickness; Mean K, mean corneal refractive power.

**Table 2:** Gender differences in the ocular and body measurements of Korean children (*n* = 165).

	Male (*n* = 64)	Female (*n* = 101)	*p*-Value
Age (years)	9.23 ± 2.06	10.06 ± 2.31	0.021 *
SEQ (D)	−2.13 ± 1.27	−2.34± 1.28	0.293
Axial length (mm)	24.70 ± 0.86	24.40 ± 0.73	0.020 *
Mean K (D)	42.89 ± 1.37	43.58 ± 1.08	0.001 *
WTW (mm)	12.02 ± 0.42	11.96 ± 0.41	0.305
ACD (mm)	3.88 ± 0.22	3.84 ± 0.21	0.312
CCT (µm)	537.44 ± 30.88	528.92 ± 31.81	0.092
Epithelium (µm)			
Central	52.70 ± 2.49	51.54 ± 3.15	0.014 *
Paracentral	52.86 ± 2.33	51.58 ± 2.99	0.004 *
Midperiphery	53.01 ± 2.16	51.81 ± 2.88	0.005 *
Stroma (µm)			
Central	484.73 ± 30.46	477.38 ± 30.96	0.136
Paracentral	504.67 ± 31.54	497.15 ± 32.23	0.143
Midperipheral	528.55 ± 33.25	521.85 ± 32.04	0.199
Height (cm)	140.39 ± 14.52	142.78± 12.31	0.259
Weight (kg)	38.10 ± 13.09	37.24 ± 9.74	0.654

* Statistically significant difference at *p* < 0.05, Student’s *t*-test. Values are presented as the mean ± standard deviation (SD). SEQ, spherical equivalent refraction; Mean K, mean corneal refractive power; WTW, white-to-white corneal diameter; ACD, anterior chamber depth; CCT, central corneal thickness.

**Table 3:** Factors affecting corneal epithelial and stromal thickness in the center in Korean school children (*n* = 165).

	Simple Regression			Multiple Regression		
	B	R	*p*-Value	B	β	*p*-Value
**Central 2 mm zone epithelium**				**0.467 (R)**		**<0.001**
Age	0.215	0.164	0.036	−0.138	−0.105	0.318
Sex	−1.159	0.191	0.014	−0.911	−0.15	0.045
Body weight	0.079	0.296	<0.001	0.095	0.356	0.001
Height	0.242	0.054	0.002			
WTW	−1.382	0.193	0.013	−1.550	−0.217	0.004
CCT	0.028	0.297	<0.001	0.019	0.198	0.008
SEQ	−0.071	0.030	0.698			
ACD	−0.937	0.068	0.388			
Axial length	0.479	0.128	0.100			
Mean K	−0.306	0.129	0.099			
**Stroma**				**0.285 (R)**		**0.003**
Age	2.403	0.175	0.025	2.715	0.198	0.011
Sex	−7.358	0.116	0.136	−10.448	−0.165	0.033
WTW	−12.106	0.162	0.037	−12.490	−0.167	0.029
Height	0.308	0.132	0.092			
Body weight	0.400	0.144	0.065			
ACD	17.84	0.124	0.114			
Axial length	4.866	0.125	0.109			
Mean K	0.276	0.011	0.877			
SEQ	−2.542	0.105	0.180			

WTW, white-to-white corneal diameter; CCT, central corneal thickness; SEQ, spherical equivalent refraction; ACD, anterior chamber depth; Mean K, mean corneal refractive power. B, unstandardized coefficients; β, standardized coefficients; R, regression coefficients.

**Table 4:** Factors affecting corneal epithelial and stromal thickness in the paracenter and mid-periphery in Korean school children (*n* = 165).

	Multiple Regression			Multiple Regression		
	B	R/β	*p*-Value	B	R/β	*p*-Value
**Epithelium**	**Paracenter**	**0.419**	**<0.001**	**Mid-periphery**	**0.367**	**<0.001**
Age	−0.047	−0.038	0.725	−0.010	−0.008	0.942
Sex	−1.135	−0.197	0.011	−1.125	−0.205	0.010
Body weight	0.072	0.295	0.008	0.055	0.229	0.036
WTW	−1.088	−0.160	0.035	−0.867	−0.134	0.084
CCT	0.014	0.154	0.043	0.010	0.113	0.147
**Stroma**	**Paracenter**	**0.301**	**0.002**	**Mid-periphery**	**0.330**	**<0.001**
Age	2.904	0.204	0.009	3.274	0.226	0.003
Sex	−10.906	−0.166	0.032	−10.566	−0.158	0.038
WTW	−14.610	−0.188	0.013	−17.061	−0.217	0.004

WTW, white-to-white corneal diameter; CCT, central corneal thickness. B, unstandardized coefficients; β, standardized coefficients; R, regression coefficients.

**Table 5:** Correlation analysis among various variables in Korean school children (*n* = 165).

	SEQ	Mean K	WTW	Axial Length	ACD	Height	Body Weight	Age
SEQ	1							
Mean K	−0.067	1						
WTW	0.061	−0.378 **	1					
Axial length	−0.634 **	−0.593 **	0.248 **	1				
ACD	−0.215 **	0.065	0.513 **	0.308 **	1			
Height	−0.332 **	−0.155 *	−0.060	0.459 **	0.174 *	1		
Body weight	−0.269 *	−0.205 **	0.118	0.447 **	0.204 **	0.869 **	1	
Age	−0.275 **	−0.031	−0.123	0.367 **	0.133	0.838 **	0.701 **	1

* Statistically significant difference at *p* < 0.05, ** *p* < 0.01. SEQ, spherical equivalent refraction; Mean K, mean corneal refractive power; WTW, white-to-white corneal diameter; ACD, anterior chamber depth.

## Abbreviations

FD-OCT	Fourier-domain optical coherence tomography
CCT	central corneal thickness
WTW	white to white corneal diameter
Mean K	mean keratometric value, mean corneal power
ACD	anterior chamber depth
SEQ	spherical equivalent refraction
